# SBGNViz: A Tool for Visualization and Complexity Management of SBGN Process Description Maps

**DOI:** 10.1371/journal.pone.0128985

**Published:** 2015-06-01

**Authors:** Mecit Sari, Istemi Bahceci, Ugur Dogrusoz, Selcuk Onur Sumer, Bülent Arman Aksoy, Özgün Babur, Emek Demir

**Affiliations:** 1 Computer Engineering Dept, Bilkent University, Ankara, Turkey; 2 Computational Biology Center, Memorial Sloan-Kettering Cancer Center, New York, NY, USA; 3 Tri-Institutional Training Program in Computational Biology and Medicine, New York, NY, USA; University of Toronto, CANADA

## Abstract

**Background:**

Information about cellular processes and pathways is becoming increasingly available in detailed, computable standard formats such as BioPAX and SBGN. Effective visualization of this information is a key recurring requirement for biological data analysis, especially for -omic data. Biological data analysis is rapidly migrating to web based platforms; thus there is a substantial need for sophisticated web based pathway viewers that support these platforms and other use cases.

**Results:**

Towards this goal, we developed a web based viewer named SBGNViz for process description maps in SBGN (SBGN-PD). SBGNViz can visualize both BioPAX and SBGN formats. Unique features of SBGNViz include the ability to nest nodes to arbitrary depths to represent molecular complexes and cellular locations, automatic pathway layout, editing and highlighting facilities to enable focus on sub-maps, and the ability to inspect pathway members for detailed information from EntrezGene. SBGNViz can be used within a web browser without any installation and can be readily embedded into web pages. SBGNViz has two editions built with ActionScript and JavaScript. The JavaScript edition, which also works on touch enabled devices, introduces novel methods for managing and reducing complexity of large SBGN-PD maps for more effective analysis.

**Conclusion:**

SBGNViz fills an important gap by making the large and fast-growing corpus of rich pathway information accessible to web based platforms. SBGNViz can be used in a variety of contexts and in multiple scenarios ranging from visualization of the results of a single study in a web page to building data analysis platforms.

## Introduction

Our knowledge about cellular processes increases at a breakneck rate, both in the axis of static process information captured in pathway models and interaction networks, and in the axis of dynamic -omic profiles that capture alterations and perturbations occurring in different contexts such as a disease or a condition. This rapid growth necessitates a parallel growth in our analysis and interpretation capabilities. A recurring component for a wide range of analysis scenarios is visualization of these networks. Due to the vast size and complexity of these networks, which often lead to the so-called hairball effect, ability to visualize and dynamically layout arbitrary sub-networks at multiple levels of detail is necessary for effective visualization. To keep up with the rapid, distributed growth of data, visualization tools should also use standard community formats such as BioPAX [1] and SBGN [2]. Seamless access to the existing pathway data corpus and -omic profile repositories and being able to overlay this data are highly desirable. Although there are existing standalone software applications that satisfy some of these requirements such as ChiBE [3] and Cytoscape [4], currently there are no web applications offering a fulfilling solution. Since most analysis tools are migrating towards web and cloud based platforms, there is an urgent need for a web based visualization tool that can meet the aforementioned requirements.

We developed a web based visualization tool named SBGNViz for effective analysis of process description maps in SBGN. SBGNViz comes in two editions: ActionScript (AS) and JavaScript (JS). Both editions use the recently developed SBGN-ML [5] file format for storing process description maps in SBGN standard. Through the Paxtools [6] library’s SBGN conversion support, SBGNViz has access to a large public pathway data corpus in BioPAX format. Various editing and highlighting options to enable focus on sub-maps and the ability to inspect genes for detailed information from EntrezGene are also offered by these tools. Full support for compound structures such as molecular complexes and cellular locations as defined in SBGN-PD are provided by SBGNViz software, using nested drawings. The latest versions also include native specialized automatic layout algorithm and complexity management techniques adapted for SBGN-PD maps.

SBGNViz is unique in that it is the only web based visualization tool for SBGN-PD maps with full support for compound structures, including a specialized compound layout capability. In addition, novel methods for reducing complexity of SBGN-PD maps without destroying the validity and integrity of processes were designed and implemented as part of SBGNViz.

## Methods

In this section, we introduce the algorithms behind the novel complexity management operations provided by SBGNViz JS edition.

**Fig 1:**
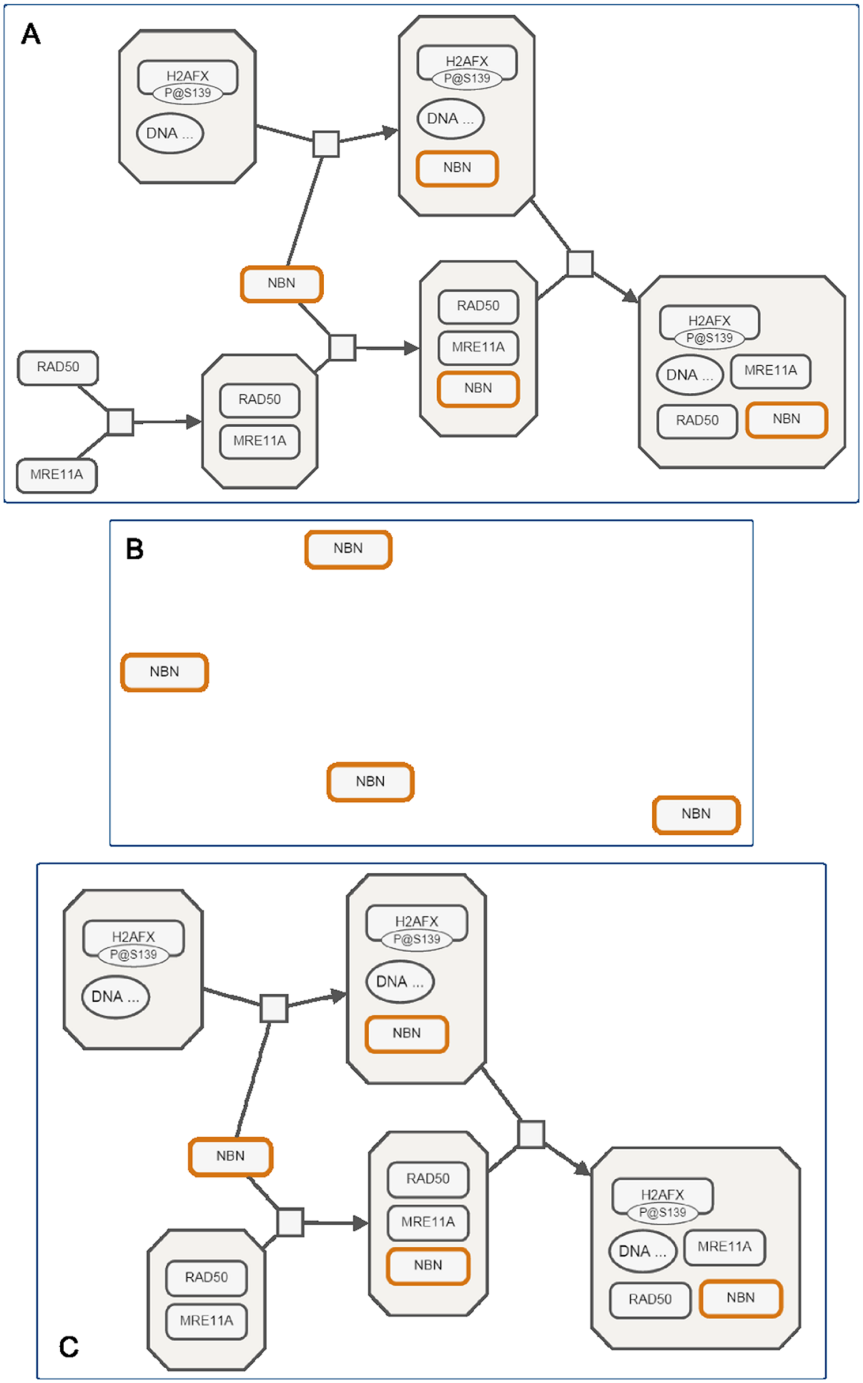
Use of generic complexity management. Application of generic complexity management techniques without the use of domain specific knowledge typically results in invalid or incomplete process description maps. (A) An SBGN map for ATM mediated phosphorylation of repair proteins in the context of MRN complex, (B) The same map after NBN instances are selected and the rest (in this case, only one of the four processes in the map) are hidden using generic filtering, and (C) The same map when proper filtering that takes domain-specific information into account is applied.

Many techniques for navigating and visualizing very large networks to reduce their complexity have been proposed in the literature [7, 8]. These range from visual methods like panning, zooming and fisheye views, to expanding and collapsing the nodes of the compound network, to hiding unwanted parts of the topology. However, application of such methods for process description maps, without the use of the domain information, hardly ever results in valid and complete maps (Fig 1).

To overcome this problem in the context of SBGN-PD maps, we keep the following invariants intact for a map, in which a specified node group is to be shown (or hidden):

- If a node is specified in a node group to be shown, it must be included.
- If a non-process node (EPN) is selected to be shown, all the processes it is involved with should be included since SBGN-PD shows the transformation of these biological entities via processes.
- If a process is selected to be shown, then all of its substrates, products, and effectors should be included as well. Hiding a substrate, product or effector might simply refer to a different process or lead to an inconsistentcy.
- If a node is selected to be shown, the parent node (a complex or a compartment) should be also included since the complexes are identified with their members and the cellular location that an entity is in might be crucial information.
- A complex molecule should always be displayed with all of its members since the member composition defines the identity of the complex.

### Focusing on a node group

We now present an algorithm that takes a group of nodes and expands this group to figure out the minimal sub-map consisting of these nodes, fulfilling the above requirements. Thus, the user can focus on specified nodes, hiding any other part of the map not directly related with the specified entities.

**algorithm** ExpandNodes(nodeGroup)

1  nodeGroup ← nodeGroup ∪ nodeGroup.descendants()

2  nodeGroup ← nodeGroup ∪ nodeGroup.parents()

3  nodeGroup ← nodeGroup ∪ nodeGroup(‘complex’).descendants()

4  processes ← nodeGroup(‘process’)

5  nonProcesses ← nodeGroup(‘!process’)

6  neighborProcesses ← nonProcesses.neighborhood(‘process’)

7  nodeGroup ← nodeGroup ∪ processes.neighborhood() ∪ neighborProcesses

   ∪ neighborProcesses.neighborhood()

8  nodeGroup ← nodeGroup ∪ nodeGroup.parents()

9  nodeGroup ← nodeGroup ∪ nodeGroup(‘complex’).descendants()

10  **return** nodeGroup

**Fig 2:**
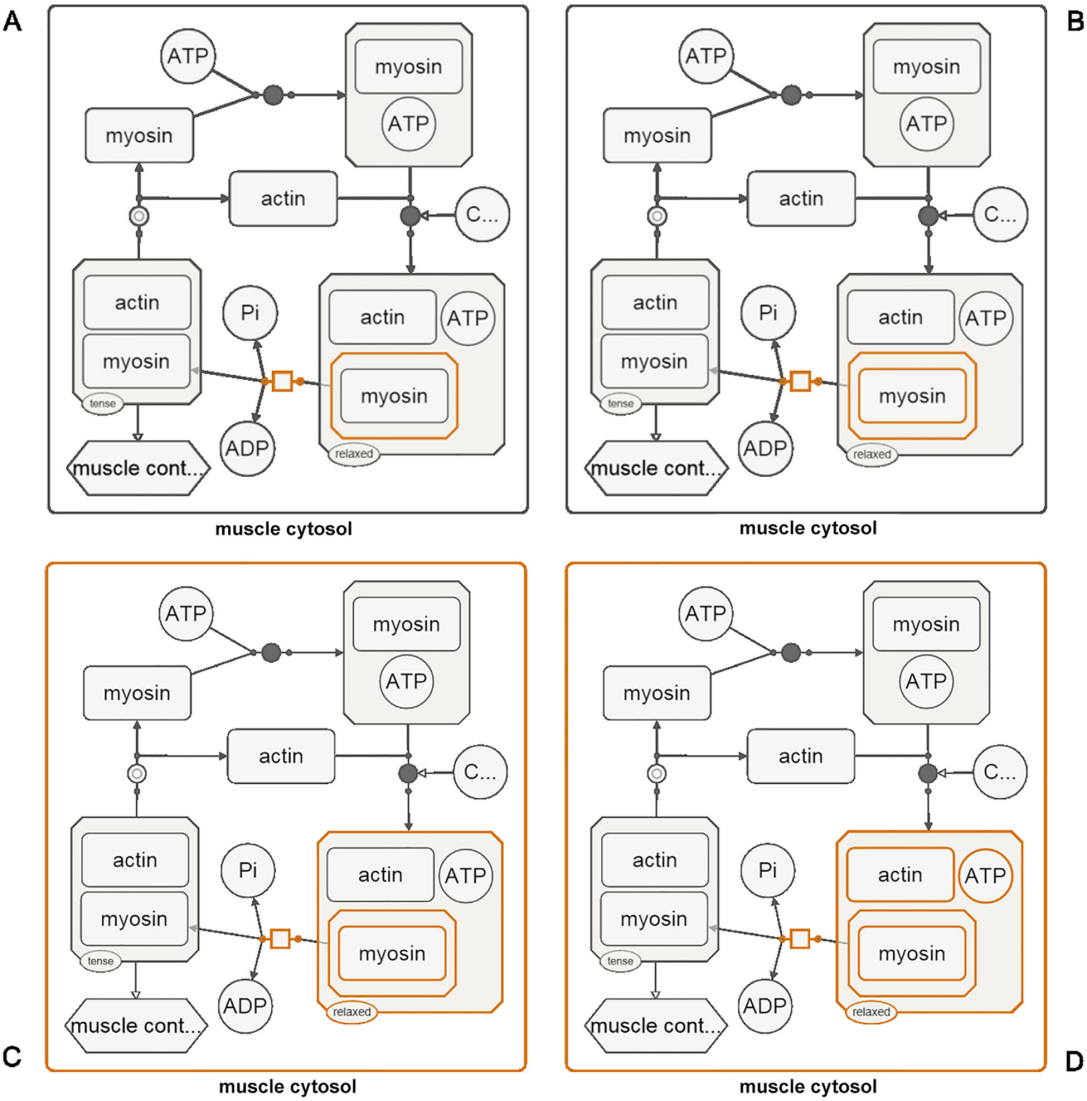
Expanding a node group. (A) A complex (containing myosin) and a process that consumes it are selected to be shown. (B) First step of the expansion algorithm adds all children of these nodes (myosin as a member of the complex in this case). (C) Then all parents of the current node group are included in the selection (a complex containing the myosin complex and muscle cytosol). (D) Node group is expanded to include any non-selected members of any complexes.

**Fig 3:**
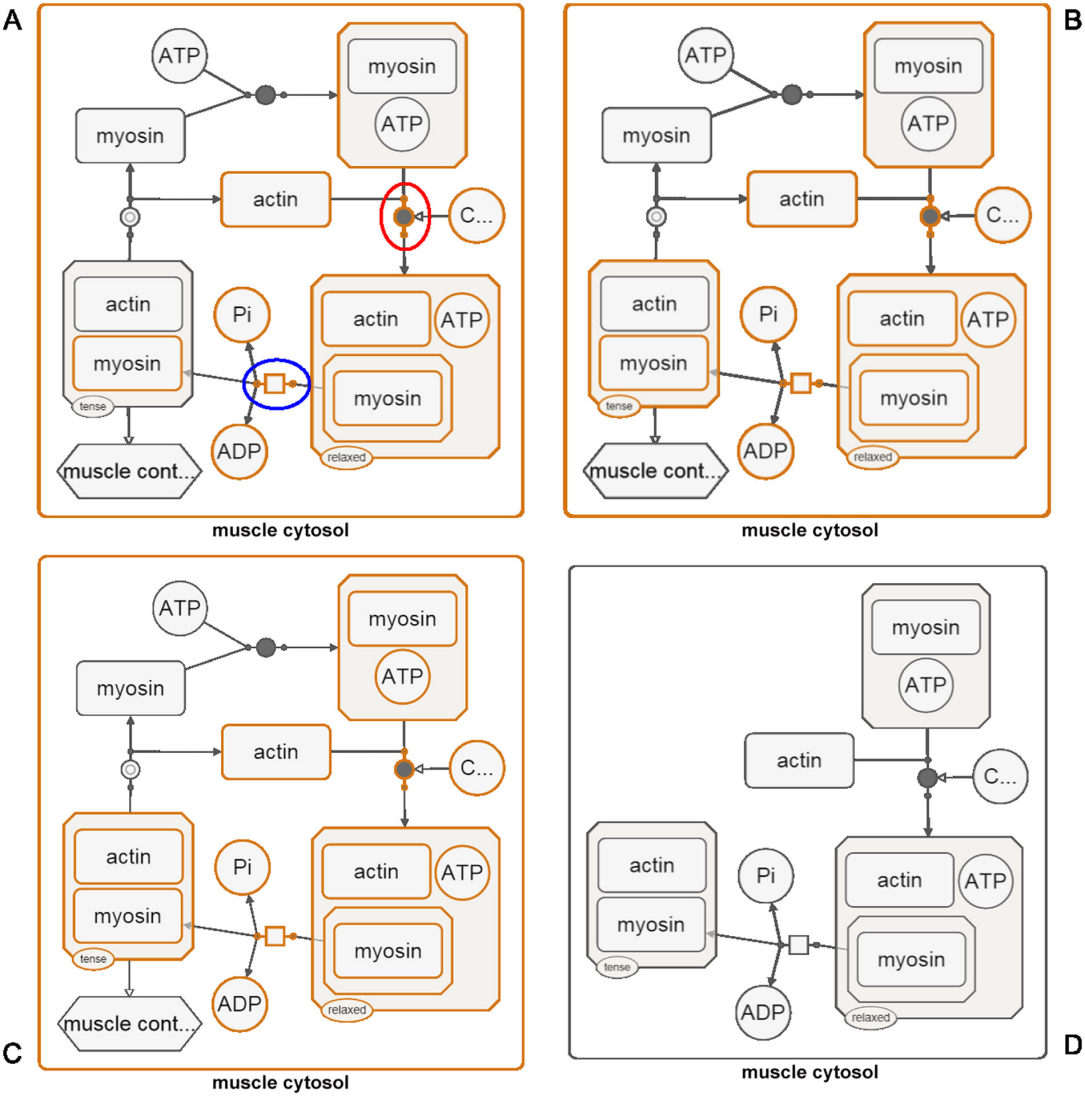
Expanding a node group (continued from Fig 2). (A) Current selection includes a process (circled in blue) and a process (circled in red) is a neighbor of the current selection. The neighboring nodes of such processes are appended to the selection. (B) Parents (e.g., actin-myosin complex) of the current selected are included. (C) Any non-selected children of selected complexes are added to the selection. (D) Resulting map includes only those processes initially selected or that the initially selected complex is involved in. The rest are hidden.

As an example, suppose we are interested in a specific process and a specific molecular complex with the protein myosin as a member in a given SBGN-PD map (those nodes selected in orange in Fig 2A). If the above expand algorithm is applied, the resulting sub-map will be a valid SBGN-PD map of reduced size (Fig 3D). The steps of algorithm ExpandNodes applied to this map are detailed in Figs 2 and 3.

### Ignoring a node group

**Fig 4:**
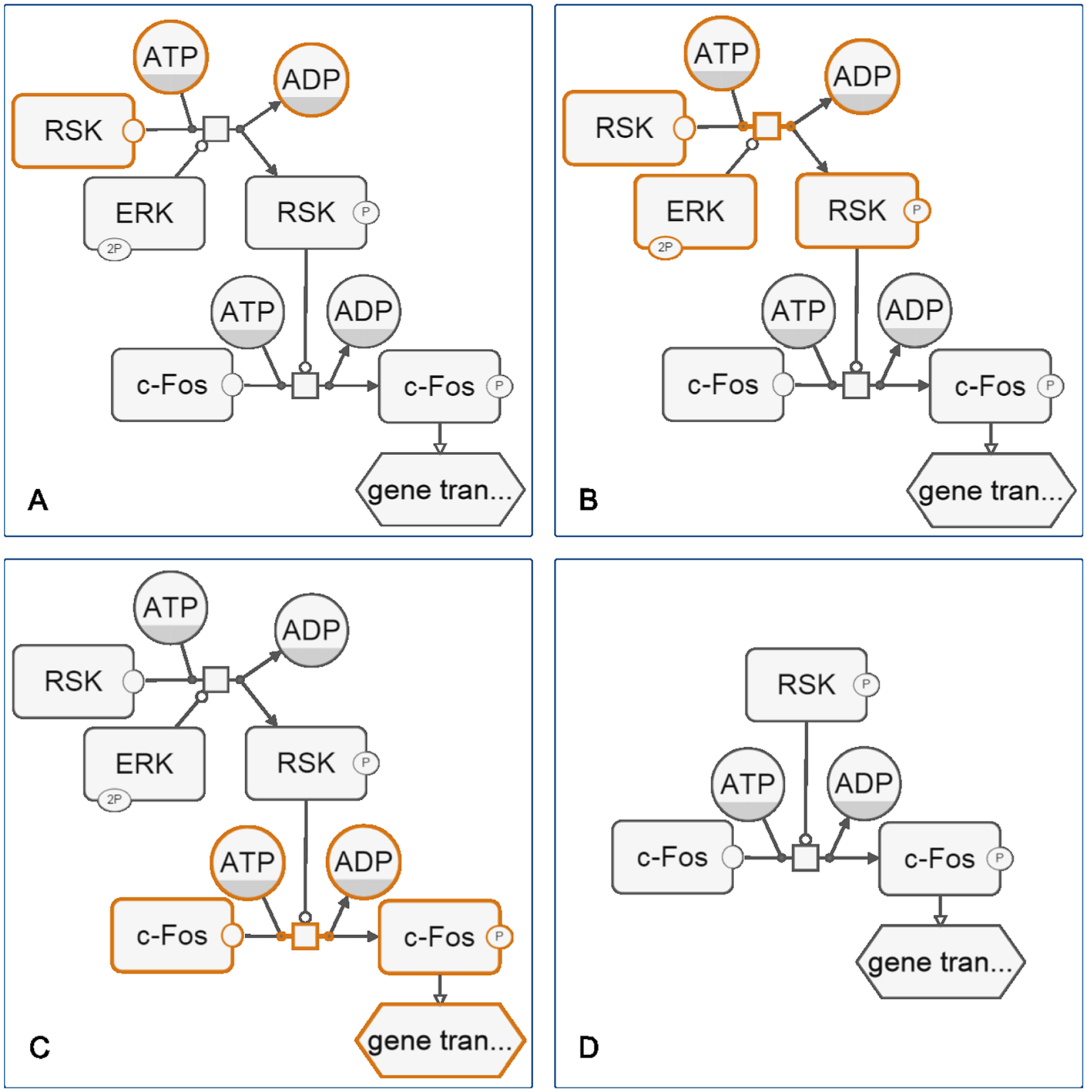
Ignoring a node group. (A) Selected nodes (in orange) are to be hidden from the drawing. (B) First, selected nodes are expanded. (C) Then, the remaining nodes (in orange) are expanded. (D) Only those nodes in the expanded group calculated in C are those nodes to be displayed.

Sometimes though, one might be interested in doing the inverse: hiding or deemphasizing certain selected entities in a map. It is natural to think that applying the expand algorithm described earlier on the remaining nodes, those not in the specified node group, should handle this situtation. However, this might undesirably expand the complement of the map to include specified nodes as well. For example, consider the map in Fig 4A. Suppose those nodes that are to be hidden are selected in orange, including native state of RSK. If we expand the remaining nodes, the native state of RSK will be included back for display; in fact, no nodes will be hidden.

The proper way to apply expansion and adjust the specified node group for the hide case is to first expand the specified nodes, followed by expanding the remaining nodes to decide on which nodes to show. The algorithm below does exactly that.

**algorithm** HideSelectedNodes(nodeGroup, allElems)

1  elemsToShow ← ExpandRemainingNodes(nodeGroup, allElems)

2  elemsToHide ← allElems.not(elemsToShow)

3  hide elemsToHide

**algorithm** ExpandRemainingNodes(nodeGroup, allElems)

1  nodeGroup ← ExpandNodes(nodeGroup)

2  remainingNodes ← allNodes.not(nodeGroup)

3  remainingNodes ← ExpandNodes(remainingNodes)

4  **return** remainingNodes

For the map in Fig 4A, hiding selected nodes in orange can thus be achieved by first expanding these nodes (Fig 4B), and then expanding the remaining nodes to end up with a sub-map (Fig 4C) to show.

## Implementation

### SBGNViz AS edition

**Fig 5:**
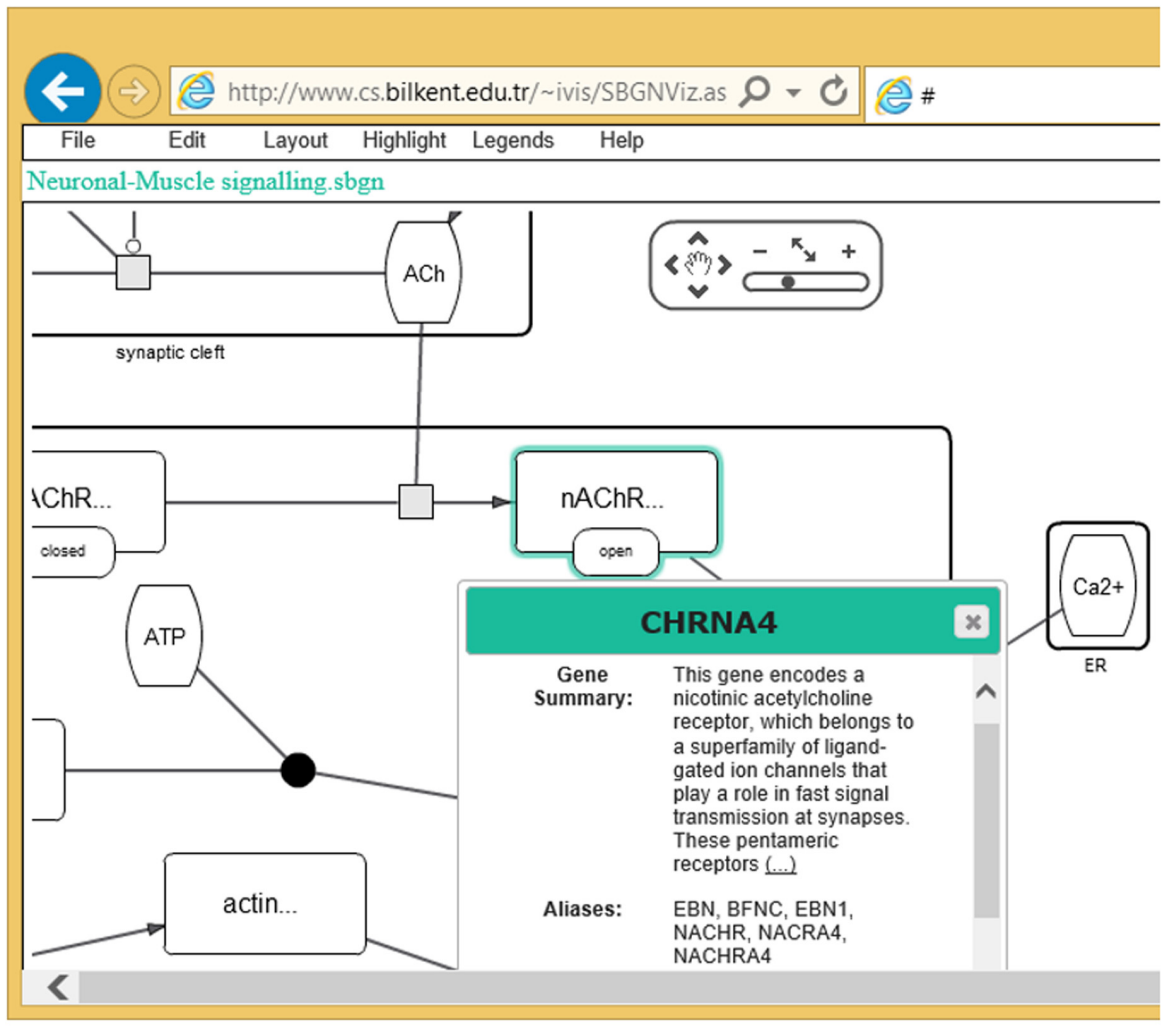
Sample screenshot from SBNViz AS edition.

SBGNViz.as is a client-side component based on Cytoscape Web [9] that requires no server side implementation, allowing developers to choose and implement any server side technology. The main network display component is implemented in Flex/ActionScript with a JavaScript API to facilitate any customization and interaction with the network view without needing to change and compile the Flash code. Even though a sample application is available to be used as an end product for visualization of SBGN-ML models (Fig 5), one can easily customize and embed SBGNViz.as within their own web pages.

**Project home page**: https://code.google.com/p/sbgnviz-as/

**Sample deployment**: http://www.cs.bilkent.edu.tr/~ivis/SBGNViz.as/

**Operating system(s)**: Web based, platform independent

**Programming language**: HTML + CSS + JavaScript + Adobe Flex

**Other requirements**: Most common browsers with recent Adobe Flash

**License**: GNU Lesser LGPL

### SBGNViz JS edition

**Fig 6:**
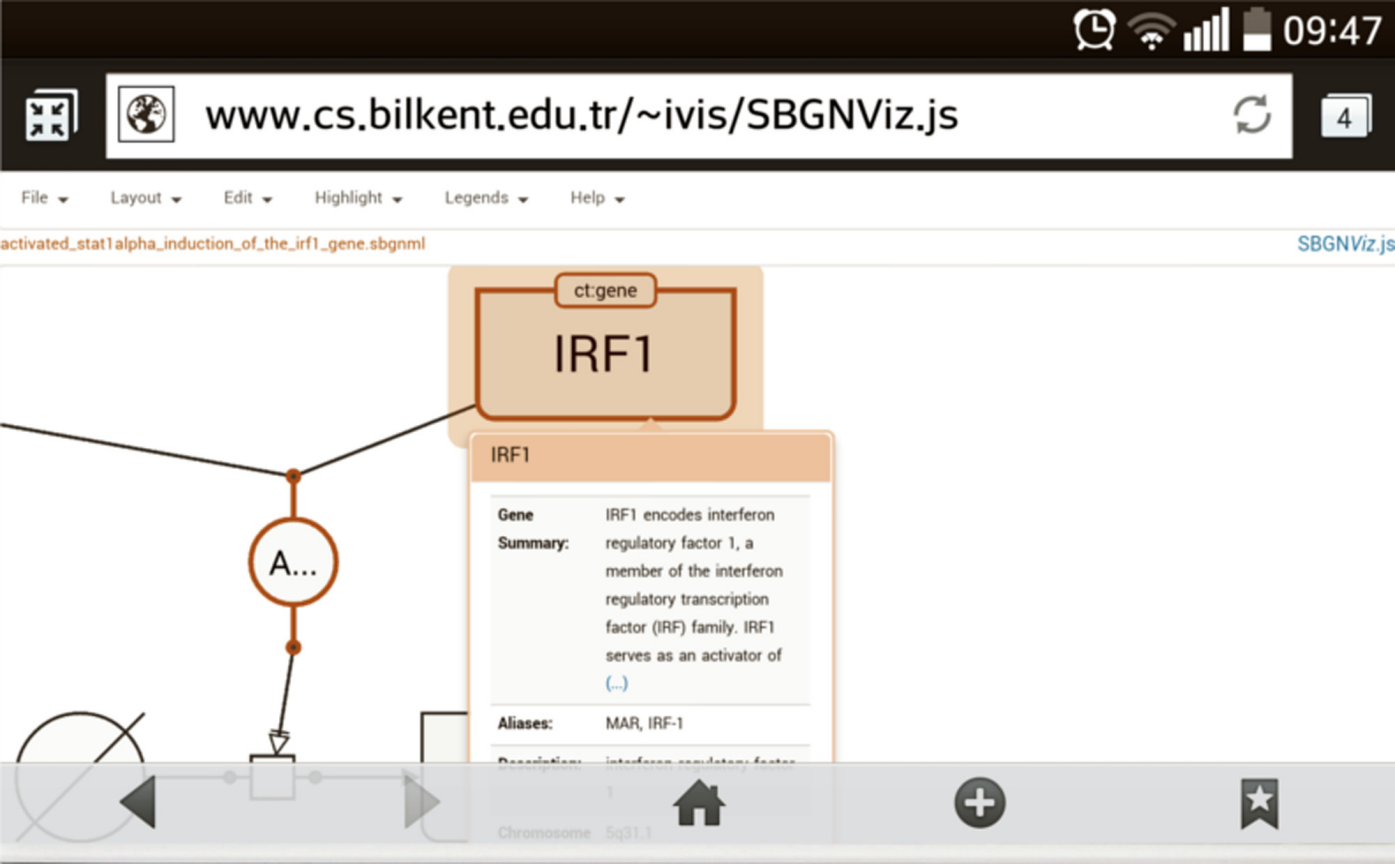
Sample screenshot from SBNViz JS edition on a smart phone.

SBGNViz.js is also a client-side component that is written purely in JavaScript based on Cytoscape.js [10]. It also comes with a sample application to be used as an end product (Fig 6) but one can easily customize and embed it within their own web pages as well.

**Project home page**: https://code.google.com/p/sbgnviz-js/

**Sample deployment**: http://www.cs.bilkent.edu.tr/~ivis/SBGNViz.js/

**Operating system(s)**: Web based, platform independent

**Programming language**: HTML5: HTML + CSS + JavaScript

**Other requirements**: Most common browsers with recent versions

**License**: GNU Lesser LGPL

## Features

### Loading and saving diagrams

Both SBGNViz editions allow input and output in SBGN-ML file format. In addition, the current drawing of the model can be saved as a static image in PNG and PDF (AS edition only) formats.

### Standard notation

Both editions of SBGNViz comply with the standard notation of SBGN-PD maps, including support for compound structures such as molecular complexes and cellular locations. Nesting structures are respected during geometry changes such as dragging of nodes in the map as well.

### Automated layout

**Fig 7:**
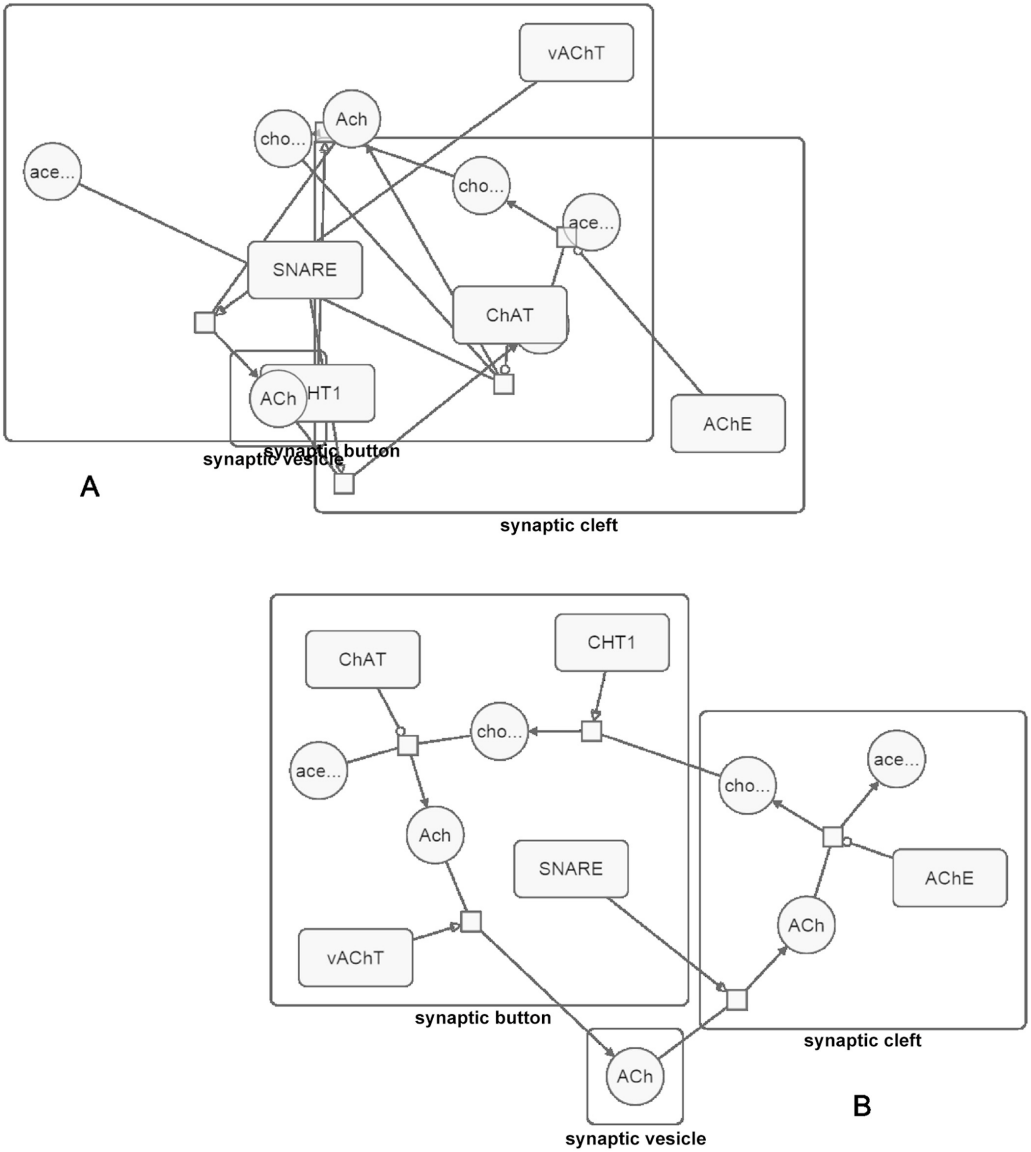
Sample layout. (A) A sample process description map laid out randomly. (B) The same map laid out using our specialized layout algorithm.

A unique feature of SBGNViz is full support for compound structures, including an automated layout algorithm handling such structures without destroying proper nesting relations (Fig 7). The layout algorithm is executed on the client side using native implementations, making it more suitable for interactive applications.

### Editing and highlighting

**Fig 8:**
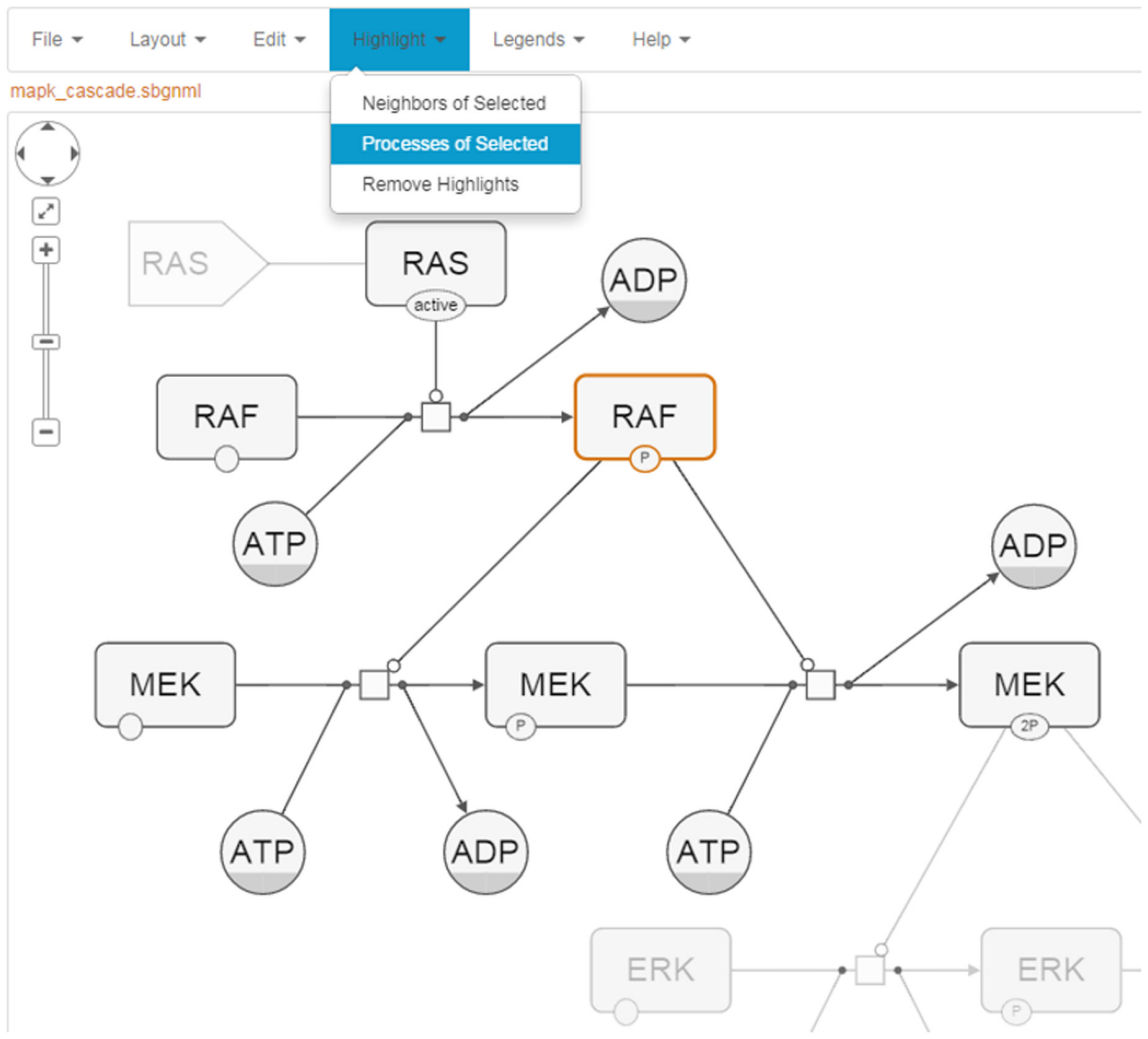
Sample highlight. A sample screenshot illustrating how selecting and highlighting processes of a selected protein works

SBGNViz AS and JS editions inherit many customary network visualization features such as zooming and scrolling, deletion of nodes and edges, and highlighting of desired portions of network (Fig 8) from Cytoscape Web and Cytoscape.js libraries, respectively, adapted for SBGN-PD maps.

### Inspection of gene details

**Fig 9:**
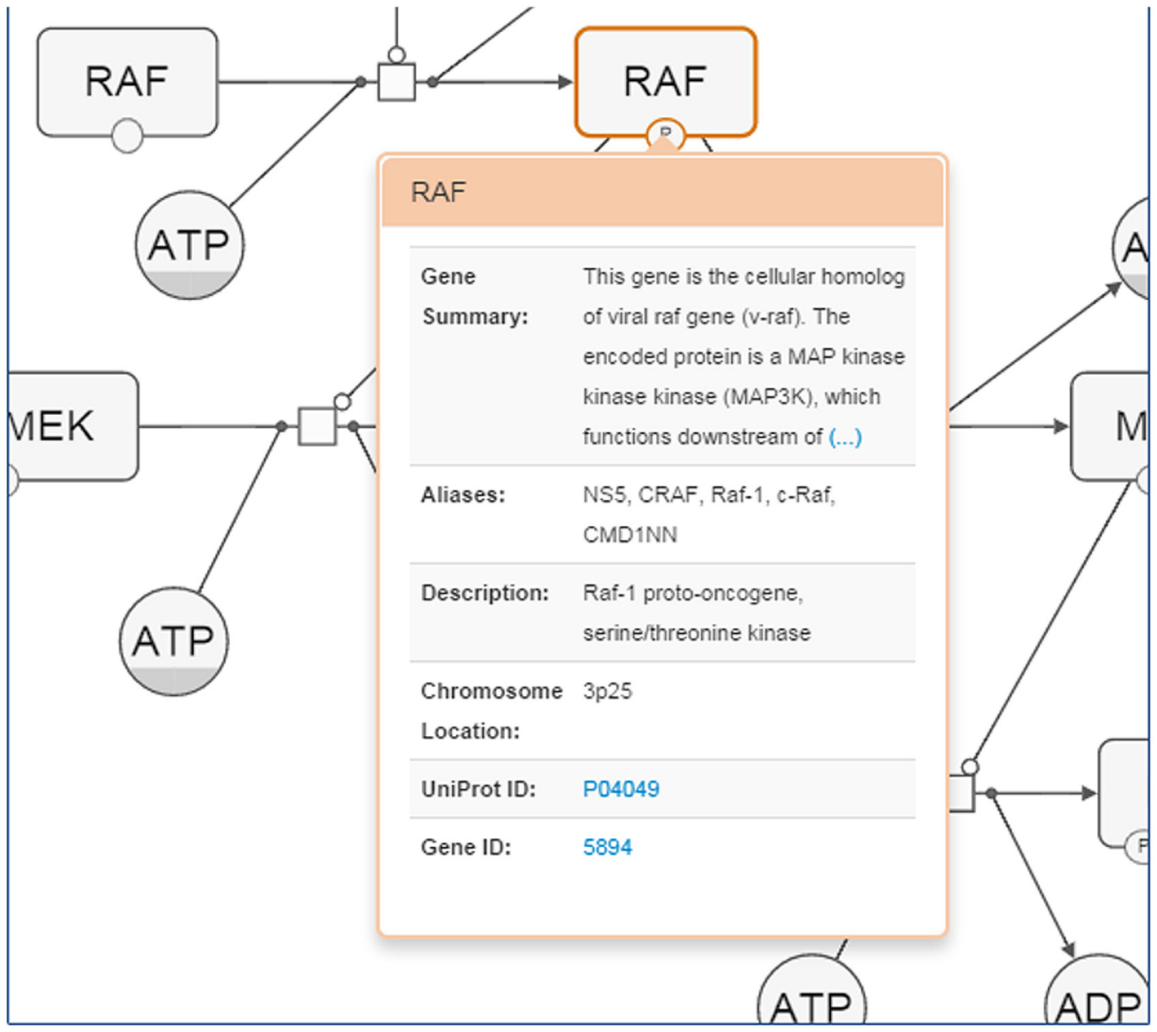
A sample screenshot illustrating how genes may be inspected for details.

Detailed properties and external references to macromolecules or nucleic acid features may be fetched from the BioGene service [11] on demand (Fig 9).

### Complexity management operations

SBGN JS edition provides operations for management of SBGN maps to reduce complexity. These operations (“Show Selected” and “Hide Selected”) are implemented using the methods described earlier.

## Conclusion

SBGNViz AS edition takes biological maps to be displayed in SBGN-ML file format and renders them in SBGN-PD notation. In addition to regular network visualization software capabilities such as zoom/scroll, highlight, and deletion of nodes/edges, the tool has full support for compound structures such as compartments and molecular complexes, including a client-side specialized automatic layout capability. Furthermore, the user is allowed to dynamically inspect gene-specific information from EntrezGene [12] through BioGene facility.

SBGNViz JS edition has similar features to the AS edition. In addition, the JS edition will work on most common browsers out of the box, without requiring any installations, and may be used on touch enabled devices such as smart phones and tablets. Furthermore, it features novel complexity management techniques for reducing complexity of SBGN maps. This enables users to focus on a part of an SBGN map without worrying about being presented with an incomplete or invalid SBGN map.

These combined features distinguish SBGNViz from tools with similar capabilities such as BioGrapher [13], SBGN-ED [14], Cell Designer [15], and CySBGN [16]. SBGNViz is unique in that it features novel methods for reducing complexity of SBGN-PD maps without destroying the validity and integrity of processes in the map. In addition, it is the only web based visualization tool for SBGN-PD maps with full support for compound structures, including a specialized compound layout capability.
